# Drug class duplication patterns among South African middle-aged adults: findings from a medicine claims database

**DOI:** 10.1007/s11096-026-02091-6

**Published:** 2026-02-24

**Authors:** Danielle Hope Fourie, Johanita Riétte Burger, Jesslee Melinda du Plessis, Martha Susanna Lubbe

**Affiliations:** Medicine Usage in South Africa (MUSA), North-West University, Potchefstroom, South Africa

**Keywords:** Drug class duplication, South Africa, Medicine claims database, Middle-aged adult, Potentially inappropriate medicine prescribing, Polypharmacy

## Abstract

**Introduction:**

Middle-aged adults (45–64 years), who constitute a large proportion of the population, are at risk of potentially inappropriate medicine (PIM) prescribing due to their high prevalence of multimorbidity. Drug class duplication, the concurrent prescription of two or more medicines from the same pharmacological class, is a commonly reported PIM criterion in studies using the PRescribing Optimally in Middle-aged People’s Treatments (PROMPT) criteria.

**Aim:**

To determine the prevalence of drug class duplication, stratified by sex and age group, and its associated factors among middle-aged adults using a South African pharmaceutical benefit management (PBM) company’s medicine claims database.

**Method:**

A cross-sectional study was conducted using data from 1 January 2023–31 December 2023. Drug class duplication was assessed across 14 pharmacological drug classes. Prevalence of drug class duplications was analysed by sex and age group, with associations tested using Pearson’s chi-square test. Spearman’s correlation coefficient (*r*_*s*_) was used to assess the correlation between drug class duplication and potential associated factors.

**Results:**

Of the 195,446 patients analysed (51.9% female; mean age 53.69 years [standard deviation (SD) 5.41, 95% confidence interval (CI) 53.665–53.713]), 48.8% experienced one or more drug class duplication. Duplication was similar between sexes (*p* = 0.1685) and higher in older age groups (*p* < 0.0001, Cramér’s* V* = 0.2). The most prevalent drug class duplications were 3-hydroxy-3-methylglutaryl coenzyme A (HMG-CoA) reductase inhibitors (statins) (n = 36,887, 18.9%); non-steroidal anti-inflammatory drugs (NSAIDs) (n = 30,829, 15.8%); angiotensin-converting enzyme (ACE) inhibitors (n = 25,646, 13.1%); angiotensin II receptor blockers (ARBs) (n = 22,411, 11.5%); calcium-channel blockers (CCBs) (n = 15,716, 8.0%); selective serotonin reuptake inhibitors (SSRIs) (n = 12,139, 6.2%); and beta-receptor blockers (β-blockers) (n = 11,577, 5.9%). Strong correlations were observed between drug class duplication and the number of Chronic Disease List (CDL) conditions per patient (*r*_*s*_ = 0.680, 95% CI 0.678–0.683), and number of prescriptions per patient (*r*_*s*_ = 0.638, 95% CI 0.636–0.641). Correlation with the number of medicine items per prescription per patient was moderate (*r*_*s*_ = 0.391, 95% CI 0.388–0.400), and weak with age (*r*_*s*_ = 0.232, 95% CI 0.227–0.236) (all *p* < 0.0001).

**Conclusion:**

Drug class duplication was common, highlighting that targeted interventions may be useful to improve patient safety.

## Impact statements

- Drug class duplication affected nearly half of middle-aged adults in this study, particularly older age groups, and was correlated with the presence of more CDL conditions and increased medicine use, highlighting that these patients may warrant prioritisation.
- Cardiovascular medicines, NSAIDs, and SSRIs were most commonly duplicated, indicating potential targets for interventions.
- Targeted interventions may be beneficial to identify and minimise inappropriate drug class duplication in this population and to promote safe and effective medicine use.

## Introduction

The safe and effective use of medicine is a fundamental principle in healthcare, yet concerns around potentially inappropriate medicine (PIM) prescribing continue to challenge patient outcomes, prompting global initiatives such as the World Health Organization (WHO) third Global Patient Safety Challenge titled “Medication Without Harm” [1]. Existing literature on PIM prescribing has largely focused on older adults ($\ge$ 65 years) due to the impact of age-related physiological changes and the high prevalence of multimorbidity (the presence of two or more chronic conditions), and polypharmacy (typically the use of five or more medicines) in this age group [2–5]. However, this emphasis may overlook safety concerns that arise earlier in the life course.

Middle-aged adults (45–64 years) are increasingly recognised as a population at risk for PIM prescribing, with a reported pooled prevalence of 38% [6]. Although the prevalence of multimorbidity increases significantly with age, a greater absolute number of individuals below the age of 65 years are multimorbid due to the larger population size of younger age groups [7]. In South Africa, multimorbidity is also common among middle-aged adults, reflecting the dual burden of chronic communicable and non-communicable diseases [8, 9]. The presence of multimorbidity often necessitates polypharmacy [10, 11], which in turn increases the likelihood of PIM prescribing, as clinical complexity can result in medicines with an unfavourable risk‐benefit profile being prescribed [3, 5, 12, 13]. Middle-aged adults represent a crucial transitional population, as intervening early in the trajectory of multimorbidity provides an important opportunity to prevent medication-related harm in later life [13, 14].

Prescribing criteria or screening tools developed to address PIM prescribing in older adults, such as the Beers criteria or Screening Tool of Older Person’s Prescriptions/Screening Tool to Alert to Right Treatment (STOPP/START) criteria, may be less applicable in middle-aged adults due to variations in age-related physiological changes and the prevalence of conditions and medicines used [14]. As such, the PRescribing Optimally in Middle-aged People’s Treatments (PROMPT) criteria were developed as the first explicit screening tool tailored for middle-aged adults [14]. The screening tool includes 22 criteria, one of which focuses on drug class duplication. This occurs when two or more medicines from the same pharmacological class are prescribed concurrently without clinical justification [14]. Such duplication can potentially increase the risk of therapeutic redundancy, adverse effects, and unnecessary healthcare costs [15–17]. Although some drug class duplications may be clinically warranted, inappropriate duplication can contribute to PIM prescribing [14–16]. Importantly, explicit criteria, such as the PROMPT criteria, are designed to be applied with little or no clinical information; however, they should not be viewed as definitive in the absence of detailed clinical data [14].

Drug class duplication is a common PIM criterion in studies in middle-aged adults, with non-steroidal anti-inflammatory drugs (NSAIDs), opioids, benzodiazepines, and selective serotonin reuptake inhibitors (SSRIs) commonly reported in previous studies [12, 13, 18]. However, there is no consensus regarding the prevalence, as findings vary considerably across studies due to differences in study design, data sources, and the demographic characteristics of the study populations [12, 13, 18]. This highlights the need for further research to better quantify and understand drug class duplication in middle-aged adults, particularly in underrepresented populations such as those in South Africa.

## Aim

This study aimed to determine the prevalence of drug class duplication in middle-aged adults using a medicine claims database from a South African pharmaceutical benefit management (PBM) company. The results were stratified by sex and age group, and potential factors associated with drug class duplication were investigated.

## Method

### Study design and data source

A cross-sectional study was conducted using secondary data obtained from a South African PBM company. The PBM company operates within the private healthcare sector of South Africa’s two-tiered healthcare system, which comprises both public and private sectors, and has been active for approximately 35 years, facilitating electronic medicine claims and managing medicine benefit administration for medical schemes. The private healthcare sector in South Africa covers over nine million beneficiaries [19], with the PBM company representing an estimated 26% of this market. The dataset included anonymised medicine claims data for 836,886 patients.

### Study population

The study population comprised 195,446 middle-aged adults (45–64 years) who had submitted at least one medicine claim between 1 January 2023 and 31 December 2023 as beneficiaries of medical schemes affiliated with the PBM company at the time of the study. An all-inclusive sample of patients meeting the selection criteria were included in the analysis. Patients with incomplete information, such as a missing date of birth, were excluded. Middle-aged adults were selected as this age group is increasingly recognised as being at risk for PIM prescribing due to their high prevalence of multimorbidity, making them a relevant population for analysing drug class duplication [12–14, 18].

### Pharmacological drug classes

The analysis of drug class duplication was guided by a criterion from the PROMPT criteria [14]. The pharmacological drug classes included in this study were benzodiazepines, non-benzodiazepine hypnotics (Z-drugs), tricyclic antidepressants (TCAs), SSRIs, opioids, NSAIDs, beta-receptor blockers (β-blockers), calcium-channel blockers (CCBs), angiotensin-converting enzyme (ACE) inhibitors, angiotensin II receptor blockers (ARBs), 3-hydroxy-3-methylglutaryl coenzyme A (HMG-CoA) reductase inhibitors (statins), stimulant laxatives, thiazide diuretics, and loop diuretics. These pharmacological drug classes were selected based on prior studies that evaluated drug class duplication using the PROMPT criteria [12, 13, 18]. Active ingredients and pharmacological drug classes were identified using the Monthly Index of Medical Specialities (MIMS^®^) [20] to include all relevant medicines available in South Africa. Fixed-dose combinations were separated into their individual active ingredients before drug class assignment to ensure accurate detection.

### Measurements

Measurements included the overall prevalence of drug class duplication and the prevalence of the individual drug class duplications, stratified by sex (female or male); and age group (45–49 years, 50–54 years, 55–59 years, and 60–64 years). Patients who received three or more prescriptions for a single medicine from any of the analysed drug classes within a six-month period were identified. Among these patients, prescribing of different medicine from the same drug class during the timeframe was identified as a drug class duplication. A six-month window was chosen as it captures multiple prescription cycles, making it more likely to identify sustained duplication rather than brief overlaps. This approach is consistent with the methodology used in a previous study [12]. All prescriptions dispensed to a patient during the study period were reviewed; however, each item meeting the criteria was only counted once, regardless of whether the same item appeared in subsequent months.

Additional measurements included the correlation between drug class duplication and age; number of prescriptions per patient; number of medicine items per prescription per patient; and number of Chronic Disease List (CDL) conditions per patient. The number of prescriptions per patient, number of medicine items per prescription per patient, and number of CDL conditions per patient served as proxies for polypharmacy and multimorbidity, given that the dataset did not permit an assessment of the total number of medicines and chronic conditions per patient in line with standard definitions. The CDL includes 26 conditions that all medical schemes in South Africa are legally required to provide coverage for, as stipulated by the Medical Schemes Act 131 of 1998 [21]. These conditions are classified as Prescribed Minimum Benefits, which aim to ensure that all beneficiaries have access to minimum healthcare services. The CDL includes Addison’s disease, asthma, bipolar mood disorder, bronchiectasis, cardiac failure, cardiomyopathy disease, chronic obstructive pulmonary disorder, chronic renal disease, coronary artery disease, Crohn’s disease, diabetes insipidus, diabetes mellitus type 1, diabetes mellitus type 2, dysrhythmias, epilepsy, glaucoma, haemophilia, hyperlipidaemia, hypertension, hypothyroidism, multiple sclerosis, Parkinson’s disease, rheumatoid arthritis, schizophrenia, systemic lupus erythematosus, and ulcerative colitis [22].

### Statistical analysis

Categorical variables were reported as frequencies and percentages, while continuous variables were reported as means with the standard deviation (SD) and 95% confidence interval (CI) for normally distributed data, or the median and interquartile range (IQR) for skewed data. The analysis of the number of CDL conditions per patient considered the total study population, including patients with zero values. In cases where the median (IQR) alone did not adequately reflect the overall trends, the mean with the SD and 95% CI were also reported. As described by the central limit theorem, in large samples, a skewed distribution does not affect the distribution of the mean [23].

Pearson’s chi-square test was used to determine associations between categorical variables, specifically the overall and individual drug class duplications, and to compare them by sex and age group. A two-tailed *p*-value of < 0.05 was considered statistically significant. Cramér’s* V* was used as a measure of effect size to assess the strength of associations between these variables (where 0.1 indicated a negligible/weak association, 0.3 a moderate association, and 0.5 a strong association) [24]. Given the skewed distribution of some variables, Spearman’s correlation coefficient (*r*_*s*_) was used to determine the strength and direction of the relationship between drug class duplication and age, number of prescriptions per patient, number of medicine items per prescription per patient, and number of CDL conditions per patient (where 0.1 indicated a weak correlation, 0.3 a moderate correlation, and 0.5 a strong correlation) [24, 25]. Analyses were performed using SAS 9.4 (TSIM3) (SAS/STAT software, Version 14.1 for Windows).

### Ethics approval

This study was approved by the North-West University Health Research Ethics Committee (NWU-00179-14-A1-16) and goodwill permission to conduct the study was obtained from the board of directors of the PBM company. To maintain anonymity and confidentiality, depersonalised medicine claims data were received from the PBM company. As such, patient consent was waived. This study was compiled in accordance with the Declaration of Helsinki.

## Results

A total of 195,446 patients were included in the study, with slightly more female than male patients (51.9% vs 48.1%) (Table 1). The mean age was 53.69 years (SD 5.41, 95% CI 53.665–53.713), and the majority of patients were in the 50–54-year age group (28.7%). A total of 2,265,558 prescriptions were analysed throughout the study period. Female patients received a higher mean number of prescriptions per patient (12.17, SD 9.24, 95% CI 12.111–12.225) compared to male patients (10.97, SD 8.65, 95% CI 10.914–11.025). There were 6,487,186 medicine items recorded, with the median number of medicine items per prescription being similar between female and male patients (2.45 [IQR 1.83–3.33] vs 2.44 [IQR 1.82–3.30]). Both the median number of prescriptions per patient and the median number of medicine items per prescription per patient increased with age, with the 60–64-year age group having the highest median number of prescriptions per patient (13 [IQR 5–18]), and median number of medicine items per prescription per patient (2.67 [IQR 1.92–3.74]). Additionally, CDL conditions were present in 80,794 patients, with a mean of 0.72 CDL conditions per patient (SD 1.06, 95% CI 0.719–0.728; median 0 [IQR 0–1]). In the 60–64-year age group, the mean per patient was 1.11 (SD 1.23, 95% CI 1.098–1.122; median 1 [IQR 0–2]), which was the highest across the age groups.

**Table 1:** Study population characteristics

Characteristic	Total study population	Sex		Age group (years)			
		Female	Male	45–49	50–54	55–59	60–64
Patients, N (%)	195,446 (100.0)	101,427 (51.9)	94,019 (48.1)	49,308 (25.3)	56,167 (28.7)	48,120 (24.6)	41,851 (21.4)
Age (years), mean (SD) (95% CI)	53.69 (5.41) (53.665–53.713)	53.76 (5.41) (53.725–53.792)	53.61 (5.40) (53.580–53.649)	–	–	–	–
Number of prescriptions^a^, n (%)	2,265,558 (100.0)	1,234,193 (54.5)	1,031,365 (45.5)	496,025 (21.8)	627,701 (27.7)	590,086 (26.1)	551,746 (24.4)
Number of prescriptions per patient^b^	11.59 (8.98) (11.552–11.632)	12.17 (9.24) (12.111–12.225)	10.97 (8.65) (10.914–11.025)	8 (3–15)	10 (4–16)	12 (4–18)	13 (5–18)
Number of medicine items, n (%)	6,487,186 (100.0)	3,540,512 (54.6)	2,946,674 (45.4)	1,284,735 (19.8)	1,724,688 (26.6)	1,737,551 (26.8)	1,740,212 (26.8)
Number of medicine items per prescription per patient, median (IQR)	2.44 (1.82–3.33)	2.45 (1.83–3.33)	2.44 (1.82–3.30)	2.30 (1.75–3.00)	2.38 (1.79–3.18)	2.5 (1.88–3.46)	2.67 (1.92–3.74)
Number of CDL conditions, n (%)	80,794 (100.0)	42,068 (52.1)	38,726 (47.9)	13,239 (16.4)	20,631 (25.5)	22,767 (28.2)	24,157 (29.9)
Number of CDL conditions per patient^b^	0.72 (1.06) (0.719–0.728); 0 (0–1)	0.72 (1.06) (0.714–0.727); 0 (0–1)	0.73 (1.06) (0.720–0.733); 0 (0–1)	0.41 (0.81) (0.406–0.420); 0 (0–1)	0.61 (0.97) (0.599–0.615); 0 (0–1)	0.84 (1.10) (0.832–0.852); 0 (0–1)	1.11 (1.23) (1.098–1.122); 1 (0–2)

CDL, Chronic Disease List; CI, confidence interval; IQR, interquartile range; SD, standard deviation

^a^Total number of prescriptions analysed throughout the study period. Patients may have multiple prescriptions and prescriptions may include multiple medicines

^b^Data are presented as mean (SD) (95% CI) and/or median (IQR)

Overall, 48.8% of patients had one or more drug class duplications (Table 2). Female patients had a slightly higher prevalence of drug class duplication compared to male patients (52.5% vs. 47.5%), although the difference in distribution was not statistically significant (*p* = 0.1685). In contrast, a statistically significant increase in drug class duplication was observed across the age groups (*p* < 0.0001); however, the practical significance was small, reflecting only a weak association (Cramér’s* V* = 0.2). The most prevalent drug class duplications were HMG-CoA reductase inhibitors (n = 36,887, 18.9%); NSAIDs (n = 30,829, 15.8%); ACE inhibitors (n = 25,646, 13.1%); ARBs (n = 22,411, 11.5%); CCBs (n = 15,716, 8.0%); SSRIs (n = 12,139, 6.2%); and β-blockers (n = 11,577, 5.9%). Female patients exhibited a statistically significant higher duplication prevalence across several drug classes, particularly benzodiazepines, non-benzodiazepine hypnotics, TCAs, and SSRIs (*p* < 0.0001 for all), although the magnitude of these associations was negligible, indicating limited practical significance (Cramér’s* V* = 0.1 for all). Duplications also increased with age for all drug classes, with the exception of stimulant laxatives (*p* = 0.5239).

**Table 2:** Prevalence of drug class duplications

Pharmacological drug class	Total study population, n (%)^a^	Sex				Age group (years)					
		Female, n (%)	Male, n (%)	*p*-value^b^	Effect size^c,d^	45–49, n (%)	50–54, n (%)	55–59, n (%)	60–64, n (%)	*p*-value^b^	Effect size^c,d^
One or more drug class duplication	95,421 (48.8)	50,117 (52.5)	45,304 (47.5)	0.1685	–	17,513 (18.4)	25,408 (26.6)	26,104 (27.4)	26,396 (27.6)	< 0.0001	0.2
Benzodiazepines	8321 (4.3)	5752 (69.1)	2569 (30.9)	< 0.0001	0.1	1523 (18.3)	2214 (26.6)	2212 (26.6)	2372 (28.5)	< 0.0001	0.1
Non-benzodiazepine hypnotics (Z-drugs)	9829 (5.0)	6415 (65.3)	3414 (34.7)	< 0.0001	0.1	1564 (15.9)	2453 (24.9)	2681 (27.3)	3131 (31.9)	< 0.0001	0.2
TCAs	4366 (2.2)	3180 (72.8)	1186 (27.2)	< 0.0001	0.1	716 (16.4)	1147 (26.3)	1177 (26.9)	1326 (30.4)	< 0.0001	0.1
SSRIs	12,139 (6.2)	8339 (68.7)	3800 (31.3)	< 0.0001	0.1	2428 (20.0)	3225 (26.6)	3180 (26.2)	3306 (27.2)	< 0.0001	0.1
Opioids	412 (0.2)	243 (59.0)	169 (41.0)	0.1423	–	60 (14.6)	118 (28.6)	98 (23.8)	136 (33.0)	0.0006	0.1
NSAIDs	30,829 (15.8)	17,236 (55.9)	13,593 (44.1)	< 0.0001	0.0	6047 (19.6)	8493 (27.6)	8465 (27.4)	7824 (25.4)	< 0.0001	0.1
β-blockers	11,577 (5.9)	5575 (48.2)	6002 (51.8)	0.0394	0.0	1516 (13.1)	2658 (23.0)	3350 (28.9)	4053 (35.0)	< 0.0001	0.1
CCBs	15,716 (8.0)	7515 (47.8)	8201 (52.2)	0.2332	–	2586 (16.5)	4054 (25.8)	4331 (27.5)	4745 (30.2)	< 0.0001	0.1
ACE inhibitors	25,646 (13.1)	11,539 (45.0)	14,107 (55.0)	0.0231	0.0	4165 (16.2)	6555 (25.6)	7261 (28.3)	7665 (29.9)	< 0.0001	0.1
ARBs	22,411 (11.5)	11,106 (49.6)	11,305 (50.4)	0.1967	–	3207 (14.3)	5564 (24.8)	6430 (28.7)	7210 (32.2)	< 0.0001	0.0
HMG-CoA reductase inhibitors (statins)	36,887 (18.9)	16,984 (46.0)	19,903 (54.0)	0.0224	0.0	4754 (12.9)	8660 (23.5)	10,712 (29.0)	12,761 (34.6)	< 0.0001	0.1
Stimulant laxatives	167 (0.1)	103 (61.7)	64 (38.3)	0.0273	0.0	33 (19.8)	50 (29.9)	39 (23.4)	45 (26.9)	0.5329	-
Thiazide diuretics	3913 (2.0)	2303 (58.9)	1610 (41.1)	0.051	–	736 (18.8)	1000 (25.6)	1130 (28.9)	1047 (26.7)	< 0.0001	0.1
Loop diuretics	3333 (1.7)	1847 (55.4)	1486 (44.6)	0.0006	0.0	389 (11.7)	675 (20.3)	1007 (30.2)	1262 (37.8)	< 0.0001	0.1

β-blocker, beta-receptor blocker; ACE, angiotensin-converting enzyme; ARB, angiotensin II receptor blocker; CCB, calcium-channel blocker; HMG-CoA, 3-hydroxy-3-methylglutaryl coenzyme A; NSAID, non-steroidal anti-inflammatory drug; SSRI, selective serotonin reuptake inhibitor; TCA, tricyclic antidepressant

^a^Percentages are based on the total study population (N = 195,446), while other percentages are based on the total of each drug class duplication

^b^Pearson’s chi-square test

^c^Cramér’s* V*

^d^Effect sizes were not included if results were not statistically significant

Correlation analyses (Table 3) revealed that drug class duplication had a strong positive correlation with the number of CDL conditions per patient (*p* < 0.0001, *r*_*s*_ = 0.680, 95% CI 0.678–0.683), and the number of prescriptions per patient (*p* < 0.0001, *r*_*s*_ = 0.638, 95% CI 0.636–0.641). A moderate positive correlation was observed for the number of medicine items per prescription per patient (*p* < 0.0001, *r*_*s*_ = 0.391, 95% CI 0.388–0.400), while a weak positive correlation was observed for age (*p* < 0.0001, *r*_*s*_ = 0.232, 95% CI 0.227–0.236).

**Table 3:** Spearman’s correlation coefficient analyses for drug class duplication

Factor	Correlation coefficient		
	*p*-value	Spearman’s correlation coefficient (*r*_*s*_)	95% CI
Age	< 0.0001	0.232	0.227–0.236
Number of prescriptions per patient	< 0.0001	0.638	0.636–0.641
Number of medicine items per prescription per patient	< 0.0001	0.391	0.388–0.400
Number of CDL conditions per patient	< 0.0001	0.680	0.678–0.683

CDL, Chronic Disease List; CI, confidence interval

## Discussion

This study investigated the prevalence of drug class duplication and its associated factors among South African middle-aged adults in a private healthcare sector medicine claims database, providing important insights into the broader issue of PIM prescribing. Drug class duplication was common, particularly in older age groups, and was correlated with the presence of more CDL conditions and increased medicine use.

Nearly half (48.8%) of middle-aged adults in this study had at least one drug class duplication. This prevalence is substantially higher than other international studies, which range from 4.3% to 11.3% [12, 13, 18]. Although direct comparisons are limited, our findings suggest that drug class duplication is a relevant concern in this population. Overall, drug class duplication was more prevalent in older age groups, consistent with existing literature indicating that these populations are at a higher risk of PIM prescribing [12, 18]. This may be attributed to the greater prevalence of multimorbidity, which can lead to polypharmacy [10, 26]. Additionally, increased health-seeking behaviour leading to more frequent healthcare encounters may also contribute [27]. Previous studies reported an association between female patients and PIM prescribing [6, 13, 18]; however, this was not observed in the present study. Several individual drug class duplications, such as benzodiazepines, non-benzodiazepine hypnotics, TCAs, and SSRIs, however, were more prevalent in female patients. These sex-related differences may reflect prescribing trends, as psychotropic medicines are more commonly prescribed to female than male patients [4, 28].

The most prevalent drug class duplications were HMG-CoA reductase inhibitors, NSAIDs, ACE inhibitors, ARBs, CCBs, SSRIs, and β-blockers. Among these, NSAID and SSRI duplication has been reported in other studies in middle-aged adults [12, 13, 18], whereas the remaining classes have not. The high prevalence of NSAID duplication may be explained by their availability over the counter in South Africa, as certain strengths and quantities are registered as Schedule 1 and 2 medicines as per the Medicines and Related Substances Act 101 of 1965 [29]. For SSRIs, their recommended use in several psychiatric disorders in South Africa [30] may contribute to the observed prevalence of drug class duplication. More broadly, fragmentation of care, such as limited care coordination between general practitioners and psychiatrists, or between different pharmacies, can compromise informational continuity [31, 32], thereby increasing the risk of drug class duplication.

Duplication within classes of cardiovascular medicines has been reported in studies involving older adults [17, 33], and was also evident in this study on middle-aged adults. High levels of duplication for medicines used to treat hyperlipidaemia, hypertension, and other cardiovascular conditions likely reflect the high prevalence of these conditions in South Africa [34, 35]. These conditions are known risk factors for cardiovascular disease, which accounts for half of all non-communicable disease deaths in South Africa [36]. Managing these conditions often involves multiple medicines [37, 38], and the widespread use of fixed-dose combinations may obscure the identity of individual active ingredients, increasing the risk of duplication [39–41]. For HMG-CoA reductase inhibitors, adverse effects often result in treatment changes, discontinuation, and subsequent reinitiation [42, 43], potentially leading to an overestimation of duplication. Benzodiazepines and opioids have also been identified as common classes involved in duplication in middle-aged adults [12, 13, 18]. However, the lower observed prevalence in this study may reflect underreporting in medicine claims data rather than actual prescribing patterns, as medical schemes may limit benefits as permitted by the Medical Schemes Act 131 of 1998 [21]. In addition, patients may intentionally avoid surveillance and pay out-of-pocket for medicines, especially if they have obtained prescriptions from multiple prescribers [44].

Drug class duplication was strongly correlated with the number of CDL conditions per patient, followed by the number of prescriptions per patient, while moderate and weak correlations were observed with the number of medicine items per prescription per patient and age, respectively. These findings suggest that drug class duplication is primarily driven by clinical complexity rather than age. Comparable associations have been reported in the literature, despite not explicitly focusing on drug class duplication [13, 45]. Moreover, this highlights the challenges of applying single-condition treatment guidelines to patients with multimorbidity, as such guidelines are often developed in isolation and rarely account for the complexities of multimorbidity, which can potentially lead to inappropriate polypharmacy and an increased risk of drug class duplication [10, 46–48].

Overall, these findings highlight that targeted interventions may be useful to identify and minimise drug class duplications, particularly among high-risk patients where clinically inappropriate duplications have been clearly identified. Interventions such as regular medication reviews and the active involvement of clinical pharmacists in the healthcare team can promote safer and more effective medicine use [49–52]. Health information technology systems, such as electronic health records (EHRs) and clinical decision support systems (CDSSs), can further support these interventions [49, 51, 52]. In the South African context, clinical pharmacists are not consistently integrated into patient care, which may be influenced by factors such as the lack of standard practice guidelines [53], while EHRs embedded with CDSSs may be constrained by factors such as infrastructure, financial, and data privacy challenges [54]. Therefore, improvements in these areas within the country may be beneficial. However, the effectiveness of most of these interventions has been determined in older adults [49, 51, 52], while research and implementation in middle-aged adults remain limited, suggesting an area for future research.

This study’s strengths include being the first known study to assess drug class duplication, specifically in middle-aged adults, in South Africa. The large all-inclusive sample, which was 23.4% of the total dataset drawn from the medicine claims database, allowed for a robust analysis of real-world data. Nonetheless, several limitations should be considered. Data-level limitations include the use of secondary data, which limited insights into clinical indications or justifications, the timing and duration of concurrent medicine use, the dose or strength of medicines used, and the use of different dosage forms. This may have led to an overestimation of drug class duplication, as not all observed duplications may be clinically inappropriate or definitive. In the absence of detailed clinical information, it was not possible to determine whether medicines were prescribed for the same or different clinical indications, or whether the results reflected true duplication or therapeutic substitution, each of which may have different clinical implications. Conversely, medicines obtained outside the claims system may result in an underestimation of drug class duplication. As polypharmacy and multimorbidity were assessed indirectly, the analysis lacks diagnostic granularity and may not fully capture the clinical complexity of patients. In addition, the findings are limited to unadjusted associations, as only bivariate analyses were performed. Furthermore, the inclusion of patients with zero CDL conditions in the calculation of the number of CDL conditions per patient may have lowered the measures of central tendency for this variable. Finally, a structural healthcare limitation of this study was that it only included data from a subset of private sector patients, potentially limiting the generalisability of the findings to the broader South African population.

Future research should explore the clinical outcomes, such as adverse drug reactions and healthcare utilisation, associated with these drug class duplications. Studies should also assess the duration of duplication to distinguish between short-term treatment transitions and prolonged duplication. Examining the dose or strength of medicines identified would further clarify whether observed duplication reflects comparable dosing or variably dosed regimens. The use of multivariable logistic regression models should be explored for the identification of independent factors associated with drug class duplication while accounting for potential confounding factors. Expanding the scope of research to include other relevant drug classes would provide further insights into duplication patterns. It is also necessary to evaluate the effectiveness of interventions used to identify and minimise drug class duplication in this population. Additionally, future research should incorporate public sector patients to provide a broader perspective on prescribing practices across the diverse healthcare settings in South Africa.

## Conclusion

Drug class duplication was common among middle-aged adults in this study, particularly for cardiovascular medicines, NSAIDs, and SSRIs. Duplication was more prevalent among older age groups, with strong correlations to the presence of more CDL conditions and increased medicine use. While some drug class duplications may be clinically justified, others may be inappropriate and present potential safety concerns. This highlights that targeted interventions may be useful to identify and minimise inappropriate drug class duplication, thereby supporting the WHO’s “Medication Without Harm” initiative by promoting safer medicine use.

## Data Availability

The authors do not have permission to share the data.

## References

[1] World Health Organization. Medication without harm: policy brief. Geneva: World Health Organization; 2023. https://iris.who.int/server/api/core/bitstreams/1eacccb6-838e-4787-bfd9-4bdeb4debfcf/content. Accessed 13 Oct 2025.

[2] Drenth-van Maanen AC, Wilting I, Jansen PAF. Prescribing medicines to older people—how to consider the impact of ageing on human organ and body functions. Br J Clin Pharmacol. 2020;86(10):1921–30. 10.1111/bcp.14094. 31425638 10.1111/bcp.14094 PMC7495267

[3] Tian F, Chen Z, Zeng Y, et al. Prevalence of use of potentially inappropriate medications among older adults worldwide: a systematic review and meta-analysis. JAMA Netw Open. 2023;6(8):e2326910. 10.1001/jamanetworkopen.2023.26910. 37531105 10.1001/jamanetworkopen.2023.26910 PMC10398411

[4] Rochon PA, Petrovic M, Cherubini A, et al. Polypharmacy, inappropriate prescribing, and deprescribing in older people: through a sex and gender lens. Lancet Healthy Longev. 2021;2(5):e290–300. 10.1016/S2666-7568(21)00054-4. 36098136 10.1016/S2666-7568(21)00054-4

[5] Lee CS, Tan NC, Goh KLS, et al. Factors associated with potentially inappropriate prescribing among older persons in primary care settings: systematic review. Proc Singap Healthc. 2023;32:20101058231181480. 10.1177/20101058231181478.

[6] Naughton M, Moriarty F, Bailey J, et al. A systematic review of the prevalence, determinants, and impact of potentially inappropriate prescribing in middle‑aged adults. Drugs Ther Perspect. 2022;38:21–32. 10.1007/s40267-021-00884-5.

[7] Barnett K, Mercer SW, Norbury M, et al. Epidemiology of multimorbidity and implications for health care, research, and medical education: a cross-sectional study. Lancet. 2012;380(9836):37–43. 10.1016/S0140-6736(12)60240-2. 22579043 10.1016/S0140-6736(12)60240-2

[8] Roomaney RA, van Wyk B, Cois A, et al. One in five South Africans are multimorbid: an analysis of the 2016 demographic and health survey. PLoS ONE. 2022;17(5):e0269081. 10.1371/journal.pone.0269081. 35617298 10.1371/journal.pone.0269081 PMC9135225

[9] Roomaney RA, van Wyk B, Cois A, et al. Multimorbidity patterns in South Africa: a latent class analysis. Front Public Health. 2023;10:1082587. 10.3389/fpubh.2022.1082587. 36711391 10.3389/fpubh.2022.1082587 PMC9875075

[10] Skou ST, Mair FS, Fortin M, et al. Multimorbidity. Nat Rev Dis Primers. 2022;8:48. 10.1038/s41572-022-00376-4. 35835758 10.1038/s41572-022-00376-4 PMC7613517

[11] World Health Organization. Medication safety in polypharmacy. Geneva: World Health Organization; 2019. https://iris.who.int/server/api/core/bitstreams/be139493-0474-49cd-9767-b72069bf42d6/content. Accessed 13 Oct 2025.

[12] Khatter A, Moriarty F, Ashworth M, et al. Prevalence and predictors of potentially inappropriate prescribing in middle-aged adults: a repeated cross-sectional study. Br J Gen Pract. 2021;71(708):e491–7. 10.3399/BJGP.2020.1048. 33606659 10.3399/BJGP.2020.1048 PMC8136579

[13] Moriarty F, Cahir C, Bennett K, et al. Potentially inappropriate prescribing and its association with health outcomes in middle-aged people: a prospective cohort study in Ireland. BMJ Open. 2017;7(10):e016562. 10.1136/bmjopen-2017-016562. 29042380 10.1136/bmjopen-2017-016562 PMC5652466

[14] Cooper JA, Ryan C, Smith SM, et al. The development of the PROMPT (PRescribing Optimally in Middle-aged People’s Treatments) criteria. BMC Health Serv Res. 2014;14:484. 10.1186/s12913-014-0484-6. 25410615 10.1186/s12913-014-0484-6 PMC4229620

[15] Jayesinghe R, Moriarty F, Khatter A, et al. Cost outcomes of potentially inappropriate prescribing in middle‐aged adults: a Delphi consensus and cross‐sectional study. Br J Clin Pharmacol. 2022;88(7):3404–20. 10.1111/bcp.15295. 35244286 10.1111/bcp.15295

[16] Schulze Westhoff M, Schröder S, Groh A, et al. Sedatives and analgesics are major contributors to potentially inappropriate duplicate prescriptions in geriatric psychiatry. Psychogeriatrics. 2023;23(2):354–63. 10.1111/psyg.12940. 36720843 10.1111/psyg.12940 PMC11578029

[17] Erhan T, Wastesson JW, Fastbom J. Trends in drug duplications in Swedish older adults: a nationwide register study from 2006 to 2021. Drugs Aging. 2024;41:775–81. 10.1007/s40266-024-01145-6. 39269595 10.1007/s40266-024-01145-6 PMC11408397

[18] Cooper JA, Moriarty F, Ryan C, et al. Potentially inappropriate prescribing in two populations with differing socio-economic profiles: a cross-sectional database study using the PROMPT criteria. Eur J Clin Pharmacol. 2016;72:583–91. 10.1007/s00228-015-2003-z. 26820292 10.1007/s00228-015-2003-z PMC4834102

[19] Council for Medical Schemes. CMS Industry report 2023. 2024. https://www.medicalschemes.co.za/the-cms-2023-industry-report-is-here/. Accessed 13 Oct 2025.

[20] Snyman JR, editor. Monthly Index of Medical Specialities (MIMS^®^). Cape Town: CTP Printers; 2023.

[21] Republic of South Africa. Medical Schemes Act 131 of 1998, as amended. Pretoria: Government Printer; 1998. https://www.gov.za/documents/medical-schemes-act. Accessed 13 Oct 2025.

[22] Council for Medical Schemes. What are prescribed minimum benefits? 2025. https://www.medicalschemes.co.za/resources/pmb/. Accessed 13 Oct 2025.

[23] Kwak SG, Kim JH. Central limit theorem: the cornerstone of modern statistics. Korean J Anesthesiol. 2017;70(2):144–56. 10.4097/kjae.2017.70.2.144. 28367284 10.4097/kjae.2017.70.2.144 PMC5370305

[24] Steyn H. Manual for the determination of effect size indices and practical significance. Potchefstroom: North-West University; 2012. https://natural-sciences.nwu.ac.za/scs/effect. Accessed 12 Oct 2025.

[25] Schober P, Boer C, Schwarte LA. Correlation coefficients: appropriate use and interpretation. Anesth Analg. 2018;126(5):1763–8. 10.1213/ANE.0000000000002864. 29481436 10.1213/ANE.0000000000002864

[26] Vos R, Boesten J, van den Akker M. Fifteen-year trajectories of multimorbidity and polypharmacy in Dutch primary care—a longitudinal analysis of age and sex patterns. PLoS ONE. 2022;17(2):e0264343. 10.1371/journal.pone.0264343. 35213615 10.1371/journal.pone.0264343 PMC8880753

[27] Ng Y, Low AJA, Chan C, et al. Healthcare utilisation patterns and contributory factors among middle-aged adults: a scoping review. J Health Popul Nutr. 2024;43:218. 10.1186/s41043-024-00715-z. 39696505 10.1186/s41043-024-00715-z PMC11657350

[28] Patwardhan V, Gil GF, Arrieta A, et al. Differences across the lifespan between females and males in the top 20 causes of disease burden globally: a systematic analysis of the Global Burden of Disease Study 2021. Lancet Public Health. 2024;9(5):e282–94. 10.1016/S2468-2667(24)00053-7. 38702093 10.1016/S2468-2667(24)00053-7 PMC11080072

[29] Republic of South Africa. Medicines and Related Substances Act 101 of 1965, as amended. Pretoria: Government Printer; 1965. https://www.gov.za/documents/drugs-control-act-7-jul-1965-0000. Accessed 13 Oct 2025.

[30] Emsley R, Colin F, Flisher AJ, et al. The South African Society of Psychiatrists (SASOP) treatment guidelines for psychiatric disorders. S Afr J Psychiatr. 2013;19(3):128–99. 10.7196/sajp.474.

[31] Prior A, Vestergaard CH, Vedsted P, et al. Healthcare fragmentation, multimorbidity, potentially inappropriate medication, and mortality: a Danish nationwide cohort study. BMC Med. 2023;21:305. 10.1186/s12916-023-03021-3. 37580711 10.1186/s12916-023-03021-3 PMC10426166

[32] Khatri R, Endalamaw A, Erku D, et al. Continuity and care coordination of primary health care: a scoping review. BMC Health Serv Res. 2023;23:750. 10.1186/s12913-023-09718-8. 37443006 10.1186/s12913-023-09718-8 PMC10339603

[33] Kim DS, Je NK, Kim GJ, et al. Therapeutic duplicate prescribing in Korean ambulatory care settings using the National Health Insurance claim data. Int J Clin Pharm. 2015;37:76–85. 10.1007/s11096-014-0042-7. 25428447 10.1007/s11096-014-0042-7

[34] Roomaney RA, van Wyk B, Turawa EB, et al. Multimorbidity in South Africa: a systematic review of prevalence studies. BMJ Open. 2021;11(10):e048676. 10.1136/bmjopen-2021-048676. 34615675 10.1136/bmjopen-2021-048676 PMC8496399

[35] Masilela C, Adeniyi OV, Benjeddou M. Prevalence, patterns and determinants of dyslipidaemia among South African adults with comorbidities. Sci Rep. 2022;12:337. 10.1038/s41598-021-04150-6. 35013433 10.1038/s41598-021-04150-6 PMC8748924

[36] Statistics South Africa. Non-communicable diseases in South Africa: findings from death notifications, 2008–2018. Pretoria: Statistics South Africa; 2023. https://www.statssa.gov.za/publications/Report-03-08-01/Report-03-08-012018.pdf. Accessed 12 Oct 2025.

[37] Tefera YG, Alemayehu M, Mekonnen GB. Prevalence and determinants of polypharmacy in cardiovascular patients attending outpatient clinic in Ethiopia University Hospital. PLoS ONE. 2020;15(6):e0234000. 10.1371/journal.pone.0234000. 32479516 10.1371/journal.pone.0234000 PMC7263581

[38] Mohsenzadeh P, Ardekani A, Poustchi H, et al. Population-based pattern of medication use and prevalence of polypharmacy among patients with cardiovascular diseases: results of the Pars cohort study from Iran. BMC Cardiovasc Disord. 2022;22:435. 10.1186/s12872-022-02872-7. 36203125 10.1186/s12872-022-02872-7 PMC9536013

[39] An J, Derington CG, Luong T, et al. Fixed-dose combination medications for treating hypertension: a review of effectiveness, safety, and challenges. Curr Hypertens Rep. 2020;22(11):95. 10.1007/s11906-020-01109-2. 33052522 10.1007/s11906-020-01109-2

[40] Choi W, Koo H, Jeong KH, et al. Therapeutic duplication as a medication error risk in fixed-dose combination drugs for dyslipidemia: a nationwide study. Korean J Clin Pharm. 2023;33(3):168–77. 10.24304/kjcp.2023.33.3.168.

[41] Moriarty F, Bennett K, Fahey T. Fixed-dose combination antihypertensives and risk of medication errors. Heart. 2019;105(3):204–9. 10.1136/heartjnl-2018-313492. 30072364 10.1136/heartjnl-2018-313492 PMC6388906

[42] Ruscica M, Ferri N, Banach M, et al. Side effects of statins: from pathophysiology and epidemiology to diagnostic and therapeutic implications. Cardiovasc Res. 2022;118(17):3288–304. 10.1093/cvr/cvac020. 10.1093/cvr/cvac020 35238338

[43] Talic S, Marquina C, Ofori-Asenso R, et al. Switching, persistence and adherence to statin therapy: a retrospective cohort study using the Australian National Pharmacy Data. Cardiovasc Drugs Ther. 2022;36(5):867–77. 10.1007/s10557-021-07199-7. 34097194 10.1007/s10557-021-07199-7

[44] Soeiro T, Lacroix C, Pradel V, et al. Early detection of prescription drug abuse using doctor shopping monitoring from claims databases: illustration from the experience of the French Addictovigilance Network. Front Psychiatry. 2021;12:640120. 10.3389/fpsyt.2021.640120. 34079478 10.3389/fpsyt.2021.640120 PMC8165176

[45] Smeaton T, McElwaine P, Cullen J, et al. A prospective observational pilot study of adverse drug reactions contributing to hospitalization in a cohort of middle‑aged adults aged 45–64 years. Drugs Ther Perspect. 2020;36:123–30. 10.1007/s40267-019-00700-1.

[46] Adan M, Gillies C, Tyrer F, et al. The multimorbidity epidemic: challenges for real-world research. Prim Health Care Res Dev. 2020;21:e6. 10.1017/S146342361900094X. 32138803 10.1017/S146342361900094X PMC7082712

[47] Ong KY, Lee PSS, Lee ES. Patient-centred and not disease-focused: a review of guidelines and multimorbidity. Singap Med J. 2020;61(11):584–90. 10.11622/smedj.2019109. 10.11622/smedj.2019109 PMC8040921 31489434

[48] World Health Organization. Multimorbidity: technical series on safer primary care. Geneva: World Health Organization; 2016. https://iris.who.int/server/api/core/bitstreams/695cc0ce-75ea-4163-8bb5-3bc9733fa63f/content. Accessed 13 Oct 2025.

[49] Kroon D, Steutel NF, Vermeulen H, et al. Effectiveness of interventions aiming to reduce inappropriate drug prescribing: an overview of interventions. J Pharm Health Serv Res. 2021;12(3):423–33. 10.1093/jphsr/rmab038.

[50] Rahayu SA, Widianto S, Defi IR, et al. Role of pharmacists in the interprofessional care team for patients with chronic diseases. J Multidiscip Healthc. 2021;14:1701–10. 10.2147/JMDH.S309938. 34267522 10.2147/JMDH.S309938 PMC8275864

[51] Rodrigues DA, Plácido AI, Mateos-Campos R, et al. Effectiveness of interventions to reduce potentially inappropriate medication in older patients: a systematic review. Front Pharmacol. 2022;12:777655. 10.3389/fphar.2021.777655. 35140603 10.3389/fphar.2021.777655 PMC8819092

[52] Santos NSD, Marengo LL, Moraes FDS, et al. Interventions to reduce the prescription of inappropriate medicines in older patients. Rev Saude Publica. 2019;53:7. 10.11606/S1518-8787.2019053000781. 30726488 10.11606/S1518-8787.2019053000781 PMC6390643

[53] Bronkhorst E, Schellack N, Gous AGS. Clinical pharmacy in South Africa: qualitative investigation of perspectives of practicing pharmacists. Afr J Pharm Pharmacol. 2022;16(10):173–82. 10.5897/AJPP2022.5329.

[54] Zharima C, Griffiths F, Goudge J. Exploring the barriers and facilitators to implementing electronic health records in a middle-income country: a qualitative study from South Africa. Front Digit Health. 2023;5:1207602. 10.3389/fdgth.2023.1207602. 37600481 10.3389/fdgth.2023.1207602 PMC10437058

